# Outcomes of Just-in-Time Patient and Stakeholder Engagement at 1 Year

**DOI:** 10.1093/geroni/igaa057.2856

**Published:** 2020-12-16

**Authors:** Carol Geary, June Eilers, Melissa Leon, James McClay, Paul Estabrooks

**Affiliations:** 1 University of Nebraska Medical Center, Omaha, Nebraska, United States; 2 UNMC, Omaha, Nebraska, United States; 3 UNMC Center for Advanced Surgical Technology, Omaha, Nebraska, United States; 4 UNMC College of Medicine, Department of Emergency Medicine, Omaha, Nebraska, United States; 5 University of Nebraska Medical Center College of Public Health, Omaha, Nebraska, United States

## Abstract

The University of Nebraska Medical Center’s Rural Patient and Stakeholder Engagement in Research project used a novel approach to patient engagement in research: just-in-time engagement. In this approach, patients were identified and matched with researchers with shared research interests without preemptive patient and stakeholder education and training. Ten research teams initiated engaged research design between August and October of 2019. In this presentation, we will share process and outcomes measures of the just-in-time approach: measures of ongoing engagement at 6 and 9 months; evidence of research productivity, and patient and stakeholder satisfaction with their research involvement. We will also address ethical challenges, including challenges to engaging a diverse population. Part of a symposium sponsored by the Patient/Person Engagement in Research Interest Group.

